# Targeted linked-read sequencing for direct haplotype phasing of maternal *DMD* alleles: a practical and reliable method for noninvasive prenatal diagnosis

**DOI:** 10.1038/s41598-018-26941-0

**Published:** 2018-06-06

**Authors:** Se Song Jang, Byung Chan Lim, Seong-Keun Yoo, Jong-Yeon Shin, Ki-Joong Kim, Jeong-Sun Seo, Jong-Il Kim, Jong Hee Chae

**Affiliations:** 1 Department of Biomedical Sciences, Seoul National University Graduate School, Seoul, Korea; 2 Department of Biochemistry and Molecular Biology, Seoul National University College of Medicine, Seoul, Korea; 3 Department of Pediatrics, Seoul National University College of Medicine, Seoul National University Children’s Hospital, Seoul, Korea; 4 Institute of Reproductive Medicine and Population, Medical Research Center, Seoul National University, Seoul, Korea; 5 Gong Wu Genomic Medicine Institute, Seoul National University Bundang Hospital, Bundang-Gu, Seongnam-Si, Kyunggi-Do Korea; 6 Genomic institute, Macrogen Inc., Seoul, Korea; 7 Genomic Medicine Institute (GMI), Medical Research Center, Seoul National University, Seoul, Korea

## Abstract

For the noninvasive prenatal diagnosis (NIPD) of X-linked recessive diseases such as Duchenne muscular dystrophy (DMD), maternal haplotype phasing is a critical step for dosage analysis of the inherited allele. Until recently, the proband-based indirect haplotyping method has been preferred despite its limitations for use in clinical practice. Here, we describe a method for directly determining the maternal haplotype without requiring the proband’s DNA in DMD families. We used targeted linked-read deep sequencing (mean coverage of 692×) of gDNA from 5 mothers to resolve their haplotypes and predict the mutation status of the fetus. The haplotype of DMD alleles in the carrier mother was successfully phased through a targeted linked-read sequencing platform. Compared with the proband-based phasing method, linked-read sequencing was more accurate in differentiating whether the recombination events occurred in the proband or in the fetus. The predicted inheritance of the *DMD* mutation was diagnosed correctly in all 5 families in which the mutation had been confirmed using amniocentesis or chorionic villus sampling. Direct haplotyping by this targeted linked-read sequencing method could be used as a phasing method for the NIPD of DMD, especially when the genomic DNA of the proband is unavailable.

## Introduction

The detection of cell-free fetal DNA (cffDNA) in maternal plasma has made noninvasive prenatal diagnosis (NIPD) more feasible and applicable in clinical settings^1^. Numerous studies using high-throughput sequencing of maternal plasma DNA have shown its reliability in detecting fetal DNA for NIPD^2–6^. In addition to the current use of NIPD for detecting aneuploidy in clinical practice^7,8^, the application of this method to monogenic diseases is being investigated^9–11^.

Previously, we demonstrated the feasibility of NIPD for DMD using the targeted capture of the *DMD* gene and massively parallel sequencing (MPS)^12^. We employed a proband-based method for resolving maternal haplotypes. This phasing information was used to determine the haplotype dosage imbalance present in maternal plasma DNA^12^. Although subsequent genetic testing in the order of proband, carrier, and fetus is the most frequently used diagnostic flow in a DMD clinic, this method cannot be performed if the genotype of the proband or other family members is unavailable^12,13^. This disadvantage can be problematic because female carriers in such instances cannot be tested before the birth of their first child.

Two recent studies have tried to overcome the abovementioned drawbacks by using microfluidics-based linked-read sequencing technology^14^ and targeted locus amplification (TLA)-based phasing^15^ to phase parental DNA directly; these studies have reported success in predicting the mutation inheritance pattern in the fetus. If successfully implemented in clinical practice, these 2 direct phasing methods may extend the clinical applications of NIPD for monogenic diseases. Although whole-genome linked-read sequencing, as reported by Hui *et al*.^14^, has the advantage of being universally applicable to multiple single-gene diseases, the combination of high-coverage (70X) whole-genome sequencing and linked-read sequencing technology may be too expensive for clinical practice. Although the targeted approach of TLA-based phasing makes it more cost-effective, the need for a new, customized target kit for NIPD may be inconvenient. Our earlier method used a single platform for targeted sequencing, which is a practical and cost-effective means of proband diagnosis, carrier detection, and NIPD^12^. Therefore, we reasoned that if the linked-read sequencing could be applied while maintaining the targeted approach without the need for any additional capture probe design, the application of NIPD could be broadened more practically.

As a proof of principle and to test the accuracy of targeted linked-read sequencing technology, we analyzed samples from 5 families affected by DMD. We show that the direct haplotyping of maternal DNA is feasible using targeted linked-read sequencing of the *DMD* region. Our targeted approach may provide a cost-efficient and feasible method for the NIPD of DMD.

## Results

### Sequencing

The deep targeted linked-read sequencing of 5 maternal gDNA samples showed relatively consistent coverage throughout the *DMD* gene, with a mean coverage yield of 676× (see Supplementary Fig. S1). A basic sequencing summary of all samples including maternal gDNA, plasma DNA, and fetal DNA, is provided in Supplementary Table S1.

N50 phase-block length, which is representative of the contiguity achieved from haplotyping, averaged 42.7 kb (range 34.6–51.8 kb)^16,17^. Although the N50 phase-block values are smaller than other whole genome linked-read sequencing studies^16,17^, the phasing results were more than adequate for subsequent analysis.

Without referring to the results from the previous study, all carrying mutations were detected from the targeted linked-read sequencing of maternal gDNA and confirmed to be consistent with those from the MPS data (Supplementary Fig. S1 and Supplementary Table S1)^12^. The number of informative heterozygous SNPs in the *DMD* region that could be used for phasing ranged from 700 to 1,000 (Supplementary Table S1).

### Direct haplotype phasing of mutant (*HapA*) and wild-type (*HapB*) allele from linked-read sequencing

**Figure 1:**
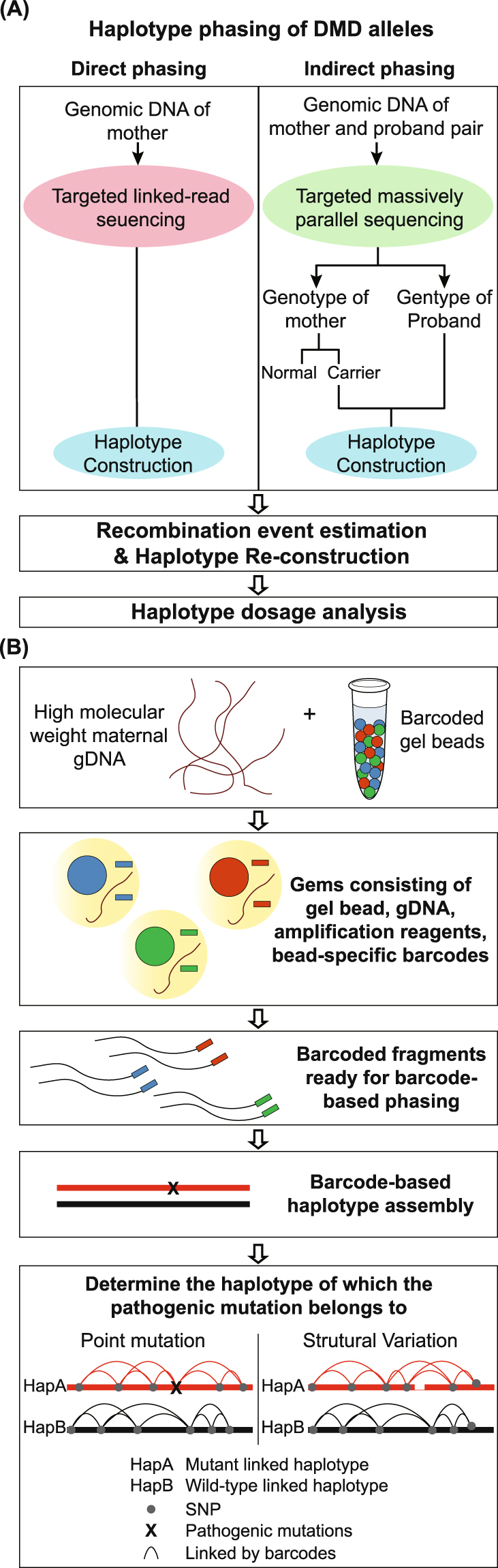
Direct haplotype phasing using targeted linked-read sequencing. (**A**) The overall workflow of phasing and subsequent analysis for noninvasive prenatal diagnosis of Duchenne muscular dystrophy. Phasing can be much simplified using targeted linked-read sequencing compared with proband-based indirect phasing. (**B**) Schematic diagram of linked-read sequencing and phasing.

**Figure 2:**
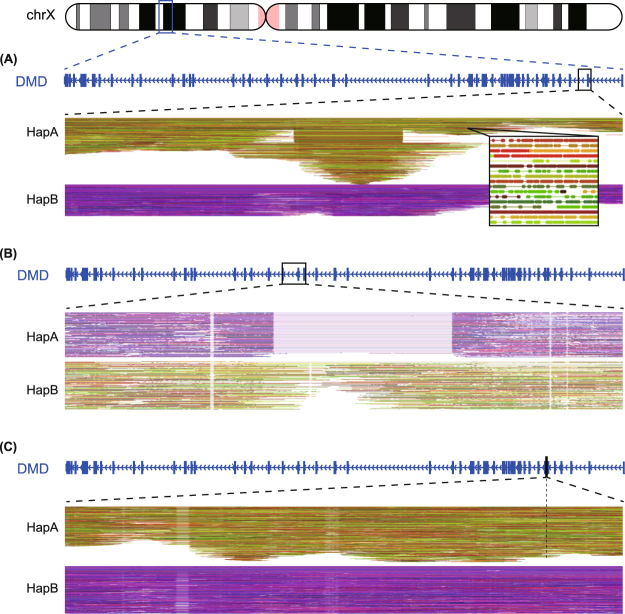
Compressed view of the linked-reads in each haplotype according to different types of *DMD* mutations. (**A**) Exon 2 duplication (DMD-02) (**B**) Exon 49–52 deletion (DMD-01) (**C**) c.649 + 2 T > C (DMD-04). Circles displayed in the magnified view represents paired-end reads, and the different colors are used for depiction of those reads that come from a single gem, sharing the same barcode.

By linking the short sequencing reads produced using the 10X genomics barcoding technology (Fig. 1B), we were able to obtain long-range information. Reads that shared the same barcode or had the same allele at heterozygous SNP positions as the mutation-supporting reads were designated *HapA*. Reads with the opposite allele at heterozygous SNP positions as the mutation-supporting reads were termed *HapB*. We directly resolved the 2 haplotypes of all 5 sets of maternal gDNA by linking the haplotype blocks assembled by the barcoded reads. Figure 2 shows examples of directly phased mutation-linked haplotypes and wild-type-linked haplotypes for different types of variations.

### Direct vs. indirect haplotype phasing and recombination event detection

**Figure 3:**
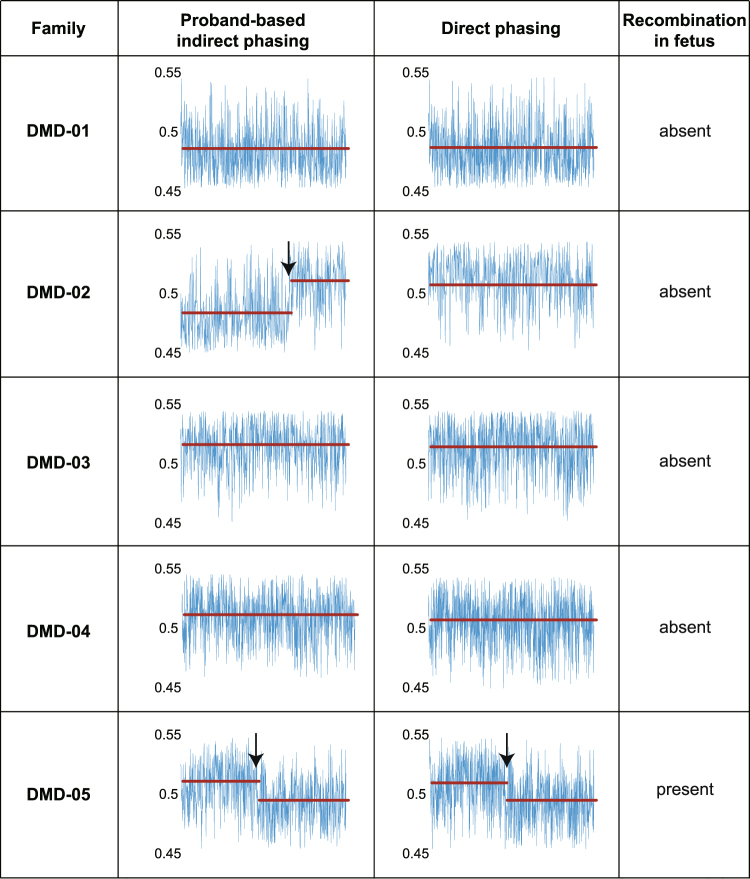
Comparison of recombination event estimation results from proband-based indirect phasing and direct phasing. The line graph represents the read fraction of the mutant allele (*HapA*) obtained from maternal sequencing data of whole *DMD* gene. The red horizontal line represents the mean read fraction of the mutant allele (*HapA*). A value greater than 0.5 indicates that the mutant allele is inherited, and an arrow at the change point indicates the possibility of recombination event. DMD-05 family was the only family with a recombination event predicted by direct haplotype phasing. Only the data from the earliest gestational weeks are displayed above (DMD-01 at 6 weeks; DMD-02 at 9 weeks; DMD-03 at 8 weeks; DMD-04 at 7 weeks; DMD-05 at 8 weeks).

Nine plasma DNA samples from 5 pregnant carriers at different gestational weeks were target sequenced in the *DMD* region. Fractional cffDNA concentrations ranged from 4.1% to 9.25% (Table 1). Before examining the haplotype imbalance between the 2 phased maternal haplotypes in plasma DNA, we investigated the recombination event within the *DMD* region. There was a significant change point in the read fraction of DMD-05 at 8 weeks (DMD-05–8-wk) and 12 weeks (DMD-05–12-wk), which represents the occurrence of a recombination event in the fetal DNA (Supplementary Figs S2A and S3E). We used the recombination point information to reconstruct the haplotypes of the DMD-05–8-wk and DMD-05–12-wk sequencing data (Fig. 3E). Concurrence between the phasing results and the fetal genotype increased in both the indirect and direct phasing methods after the recombination event adjustment (Fig. 3E and Supplementary Table S2).

**Table 1:** DMD mutation status of the study cohort.

Study Number	Genotype of Mother	Predicted Genotype of Fetus^a^	Gestational Age	Fetal DNA Concentration (%)
DMD-01	Exons 49–52 deletion/Normal	Normal	6 weeks 5 days, 17 weeks 1 day	5.66, 7.74
DMD-02	Exon 2 duplication/Normal	Exon 2 duplication	9 weeks 3 days, 12 weeks 1 day	9.25, 6.85
DMD-03	Exons 3–7 deletion/Normal	Exons 3–7 deletion	8 weeks 5 days, 11 weeks 3 days	6.34, 8.80
DMD-04	c.649 + 2 T > C/Normal	c.649 + 2 T > C	7 weeks 1 day	6.24
DMD-05	Exons 52–62 deletion/Normal	Exons 52–62 deletion	8 weeks 2 days, 12 weeks	4.10, 5.07

^a^All fetuses were male.

Interestingly, in our previous analysis, using the proband-based haplotype phasing approach, the fetus of the DMD-02 family was predicted to have a recombination event that had to be corrected before estimating the dosage imbalance (Fig. 3B and Supplementary Fig. S4B)^9,12^. However, the direct phasing approach using linked-read sequencing showed that this recombination event in fact had occurred in the proband rather than in the new fetus (Fig. 3B and Supplementary Fig. S4A). This clearly indicates that haplotype phasing using linked-read sequencing is both simpler and more accurate for distinguishing whether the recombination occurred in the fetus or in the proband. The direct phasing results in all 5 samples were >90% concurrent with the fetal genotype (Supplementary Table S2). No recombination events were detected in DMD-01, DMD-03, or DMD-04 (Supplementary Figs S2 and S3A,C,D).

We predicted the fetal genotype by resolving the allele fraction imbalance between the 2 haplotypes in the maternal plasma. The predicted results were all correct when compared with the fetal genotype. Detailed results are shown in Supplementary Fig. S3.

## Discussion

In this study, we improved on our previous method of NIPD for DMD^12^ by directly phasing the maternal haplotype using linked-read sequencing. Proband-based indirect haplotype phasing involves complex computational steps and requires the DNA of the affected male proband^12,18^. NIPD using targeted linked-read sequencing has the advantage of requiring neither the genomic data of a proband or other family members to predict the fetal mutation status accurately nor an additional capture probe. The carrier mother can therefore be tested during her first pregnancy without collecting genetic information from other family members. This new approach provides a more efficient method that can be incorporated into genetic counseling and diagnosis, as well as a more cost-effective alternative to other NIPD methods.

The inheritance of mutant-linked maternal alleles can be estimated only by comparing the dosage between the mutant and wild-type linked alleles because of the high background of maternal DNA. This means that recombination event adjustment for dosage imbalance detection is critical. The proband-based haplotyping method cannot distinguish whether the recombination event occurred in the proband or in the fetus, which increases the number of recombination adjustments needed. For example, in our previous study^12^, DMD-02 was interpreted as having a recombination event, but did not show any recombination point using the current direct haplotyping method, which suggests that this recombination occurred in the proband. Although the fetal genotype could still be predicted correctly regardless of the timing of the recombination event, any increase in the number of recombination adjustments inevitably may increase the number of computational errors. Although the larger amount of data should be added, the direct haplotyping method from linked-read sequencing has a clear advantage in recombination analysis.

Compared with the methods used in the 2 recent studies that have introduced the direct haplotyping method for the NIPD of monogenic diseases, our method has additional advantages. Although the whole-genome-based haplotyping method of Hui *et al*.^14^ can be applied to nearly all monogenic diseases, and has advantages in the prediction of recombination events, this method is too costly for clinical applications. The targeted locus amplification approach of Vermeulen *et al*.^15^ is less expensive than the whole-genome-based method, but customization of the target region may be more complex because the population frequency of SNPs may differ with ethnicity. In addition, recombination adjustment is difficult with this method. Thus, in the event of recombination, the result would be either inconclusive or falsely predictive. Further, these 2 methods require separate capture and sequencing platforms for proband diagnosis, carrier detection, and maternal plasma DNA sequencing. We believe that our targeted linked-read sequencing-based haplotyping method has advantages over the other 2 direct phasing methods in terms of its recombination prediction and cost-effectiveness. Because linked-read sequencing can also accurately detect large deletions and duplication mutations in *DMD*, this method could be used for carrier diagnosis as well as for NIPD. Haplotype information obtained from the same sequencing data could be used for future NIPD.

Although our approach has advantages over 2 recently introduced direct phasing methods, the cost effectiveness in a real clinical practice should be addressed. The major advantage of our method in terms of the cost is that the proband DNA does not need to be sequenced, because the current NIPD of DMD in practice requires 3 samples including that of the proband^19^. This reduction in cost will offset the cost of the expensive library preparation step in linked-read sequencing. The estimated laboratory cost of NIPD for 1 DMD family with our custom capture probe would be about 2,300 US dollars in both proband-based and direct phasing methods (Supplementary Table S3). Multiplexing of a barcoded library from linked-read sequencing is also feasible and will decrease the cost further. Because linked-read sequencing requires the additional step of library construction, the turnaround time would be 3 weeks, which is longer than that for the proband-based method but is still affordable for NIPD.

We admit that this approach is best suited for the NIPD of DMD. The application of this targeted approach to other monogenic diseases should be demonstrated separately. Designing the target region and capture probe is crucial for successful implementation. Although no recommended guideline currently exists, Lam *et al*.^10^ suggested the number of SNPs (1000) and sequencing depth (200-fold) by computational simulation that could be used confidently for relative haplotype dosage analysis, even with a low concentration of fetal DNA. Recombination hot spots around the target region must be checked and included in the recombination adjustment.

Since very few studies have addressed the clinical applicability of linked-read sequencing data to NIPD, more research is necessary to verify the effectiveness and readiness of this technique. Nevertheless, our direct haplotyping approach using a targeted linked-read sequencing platform illustrates a clear advantage over proband-based indirect haplotyping and could provide extended opportunity for NIPD of DMD.

## Methods

### Sample collection

The targeted sequencing data of probands, fetuses, and carrier mothers (DMD-01~04) were used from a previous study^12^. Genomic DNA from the proband, fetus, and carrier mother from 1 additional family, DMD-05, was sequenced as reported in the previous study^12^. Targeted linked-read sequencing was performed on the carrier mother’s gDNA (DMD-01~05). All 5 families had different mutations in *DMD* (Table 1).

### Linked-read sequencing

The overall workflow of our study is shown in Fig. 1A. First, we obtained gDNA from the blood cells of the 5 carrier mothers. Next, we used maternal high-molecular weight gDNA (average 52.7 kb) to acquire barcoded DNA molecules, using the 10X Genomics Chromium^TM^ library (Pleasanton, CA) (Fig. 1B). The 10X technology uses a microfluidic device to partition each genomic DNA into individual oil-enclosed gel beads, or *gems*. Every fragment of the same gem is tagged with unique, distinguishable barcodes to create a genetic library environment within a single gem^20^. We then performed targeted linked-read sequencing on all 5 carrier mothers. The barcoded reads from the above were captured using the same customized probe kit as in the previous study^12^. The barcoded and enriched reads were then sequenced using an Illumina HiSeq. 2500 sequencing system (San Diego, CA).

### Direct haplotype phasing and variant calling

The maternal haplotype in the *DMD* region was directly resolved by linking the barcoded sequence reads linearly using the freely available *Long Ranger* (v.2.1.2) software. We used the “wgs” option when running *Long Ranger* because the design of our target capture probe includes both the exonic and the intronic regions of *DMD*. The barcoded reads were then aligned to the human genome (GRCh37/hg19) using 10X Lariat^TM^. Reads with the same barcode information came from the same original long input DNA, which enabled us to link the reads to the formation of large haplotype blocks (Fig. 1B)^20^. Variant calling was performed using the FreeBayes method in *Long Ranger*. Heterozygous single nucleotide polymorphisms (SNPs), linked to either the haplotype with the mutant allele or the wild-type allele, were used in subsequent analyses for fetal genotype prediction and recombination detection. LUMPY was used to detect precise structural variation breakpoints^21^. We confirmed that these results accorded with our previous results obtained from targeted MPS^12^. Since large deletion/duplication mutations in *DMD* gene can be a hindrance to haplotype phasing in linked-read sequencing, we added a step to confirm the linkage between large deletion/duplication mutations and the phased haplotypes. For large deletions, we first defined the reads with the same barcode as the wild-type haplotype and those reads that did not share the same barcodes as the wild-type haplotype were considered the mutant haplotype. Next, we re-aligned the *HapA* reads to a customized deletion reference. We confirmed that the reads align properly to the deletion reference and that the heterozygous SNPs at the 5′ end and the 3′ end of the deletion belong in the same haplotype. For large duplications, we selected the reads with particularly divergent allele frequencies between reference and alternate alleles and examined the reads that belong to the same gem as such reads.

### Fetal genotype prediction

Because chromosome X is prone to high recombination rates, tests to detect a recombination event are imperative for accurately predicting the fetal genotype. The R package “qcc” was used to remove outliers and prevent errors in predicting the recombination point caused by outlier values from duplicated or repetitive sequences^22^. We then used the R package “changepoint” to predict the statistically accurate change point in the read fraction values^23^. The fetal genotype prediction was measured after recombination event adjustment (Fig. 1A). The fractional fetal DNA concentrations and fetal genotype predictions were measured using the method described in our previous study^12^. The institutional review board approved the study protocol (IRB no. 1606-017-768).

## Electronic supplementary material

Supplementary Information
