# Supplementary material for: Targeted linked-read sequencing for direct haplotype phasing of maternal DMD alleles: a practical and reliable method for noninvasive prenatal diagnosis
Source: Sci Rep. 2018 Jun 6;8:8678. doi: 10.1038/s41598-018-26941-0 (PMC5989205; doi:10.1038/s41598-018-26941-0)
Supplement: Supplementary file 1 — Supplementary Information [file 41598_2018_26941_MOESM1_ESM.pdf]

Supplementary Information

Targeted linked-read sequencing for direct haplotype phasing of maternal DMD alleles: a practical and reliable method for noninvasive prental diagnosis

Se Song Jang, Byung Chan Lim, Seong-Keun Yoo, Jong-Yeon Shin, Ki-Joong Kim, Jeong-Sun Seo, Jong-Il Kim, and Jong Hee Chae

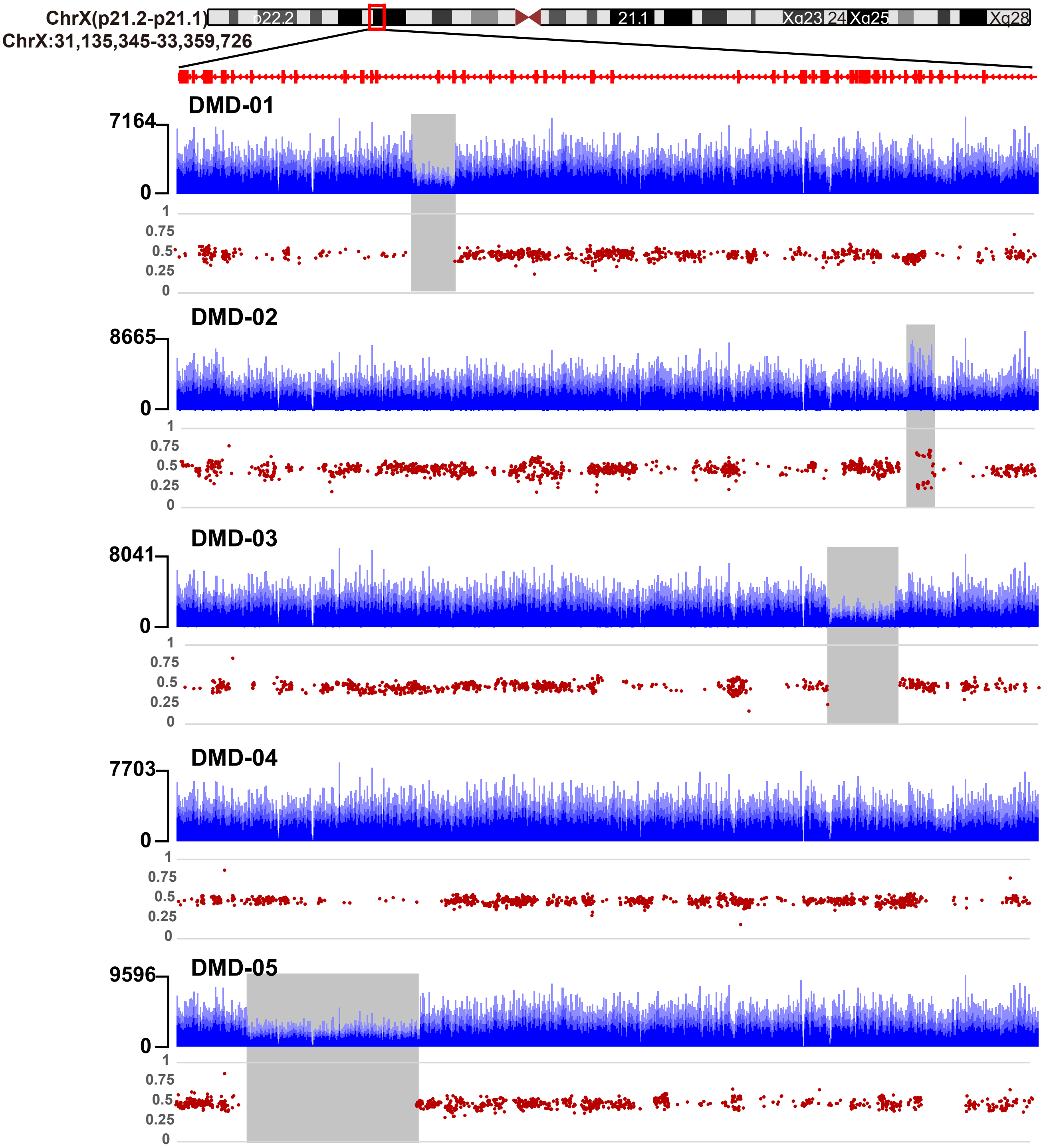

Supplementary Figure S1. Coverage and allele frequency plots of maternal genomic DNA

The red vertical bars in the graph at the very top represent the 79 exons in the DMD gene.The blue bars indicate the coverage depths of DMD. The dark red dots represent allele frequencies of heterozygous SNPs of maternal genomic DNA samples. The pathogenic mutation region in DMD gene is highlighted in grey. The deletion regions in DMD-01, 03, and 04 do not consist of any heterozygous SNPs. In the duplication region of DMD-02, with the increase in copy number, the allele frequency values cluster around 1/3 and 2/3.

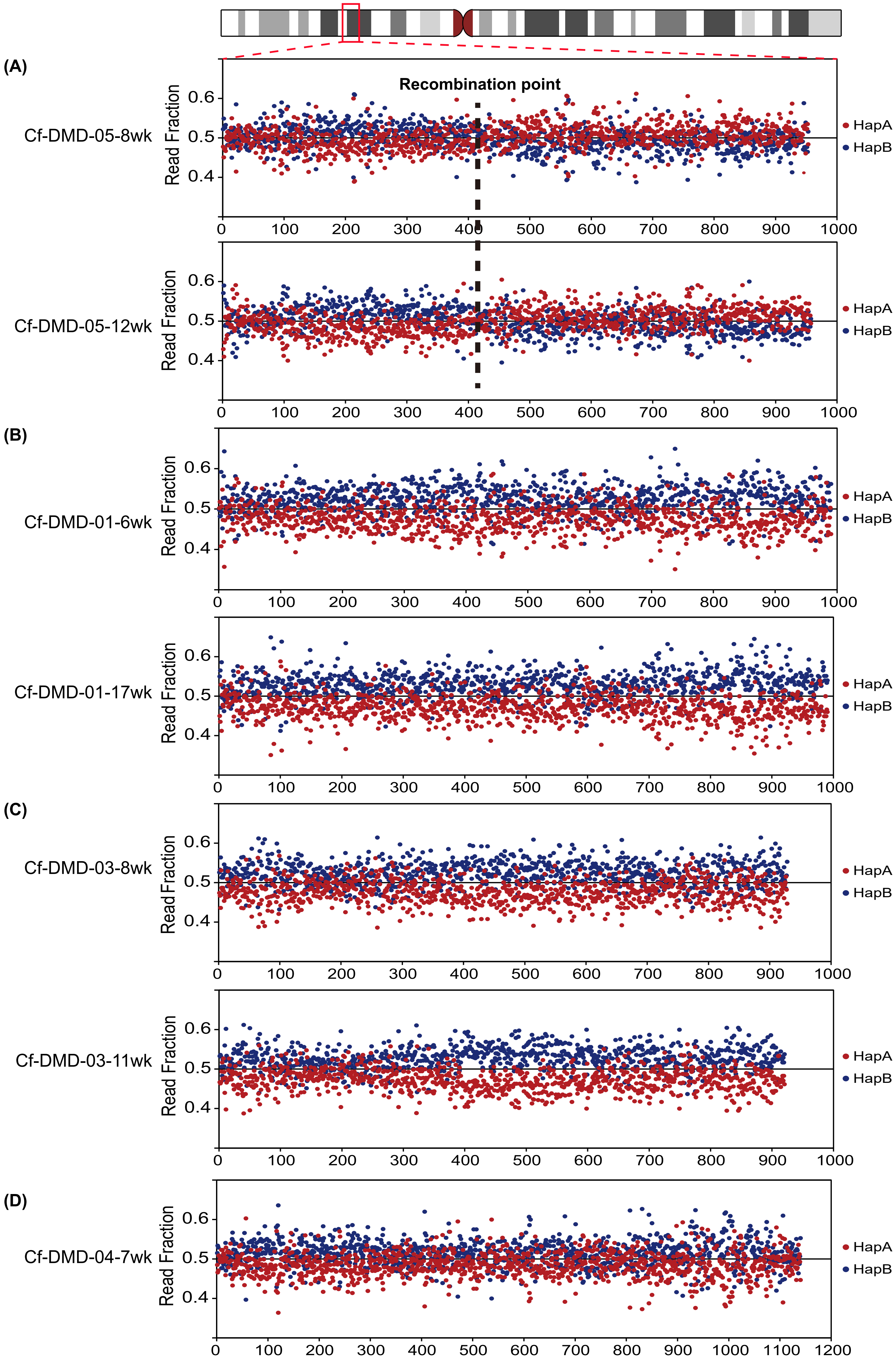

Supplementary Figure S2. Allele fraction distribution of plasma samples

(A). DMD-05, (B). DMD-01, (C). DMD-03, (D). DMD-04

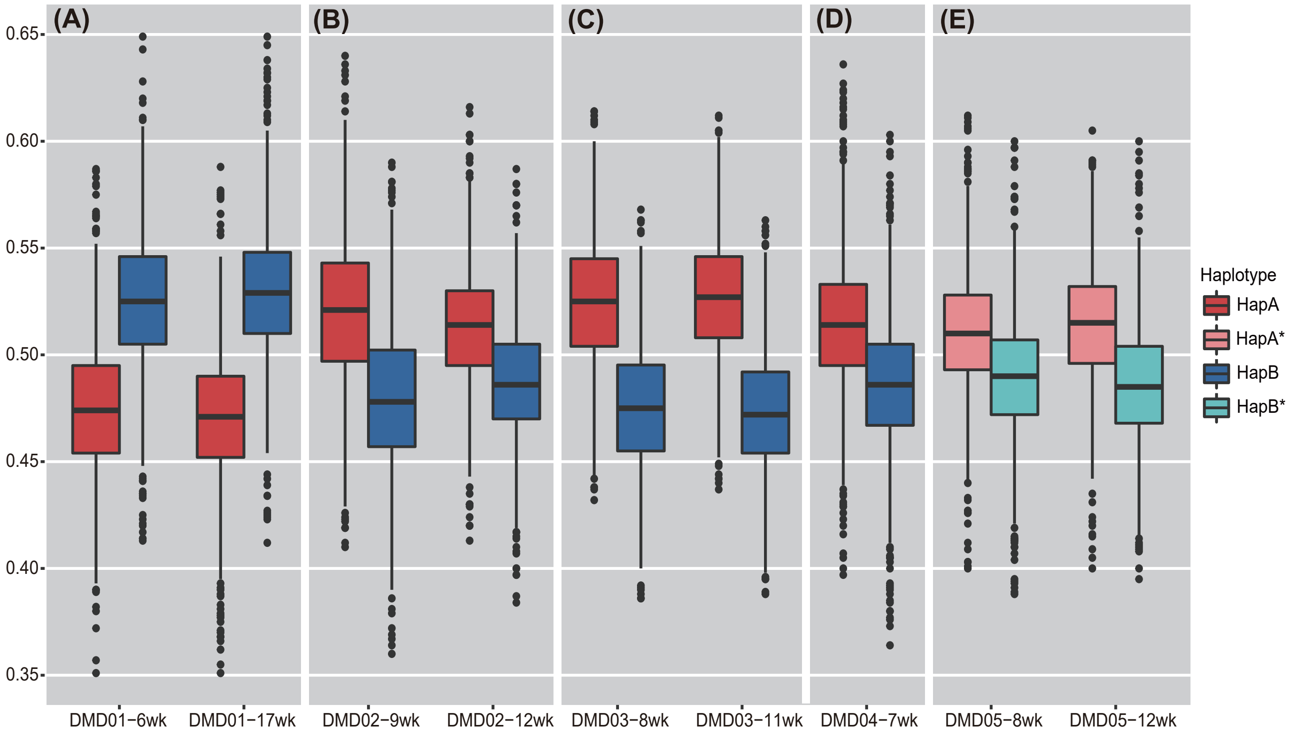

Supplementary Figure S3. Fetal genotype prediction

HapA represents the mutant-linked allele and HapB represents the wild-type-linked allele. After the detection of the recombination events, we reconstructed the haplotypes and designated them as HapA* and HapB*, where HapA* represents the recombination adjusted mutant-linked allele and HapB* the wild-type allele. HapB is overrepresented in DMD-01 maternal plasma samples. HapA or HapA* are overrepresented in rest of the maternal plasma samples. Allele fraction differences in all 5 samples were significant ($P < 0.001$).
A. DMD-01, B. DMD-02, C. DMD-03, D. DMD-04, E. DMD-05.

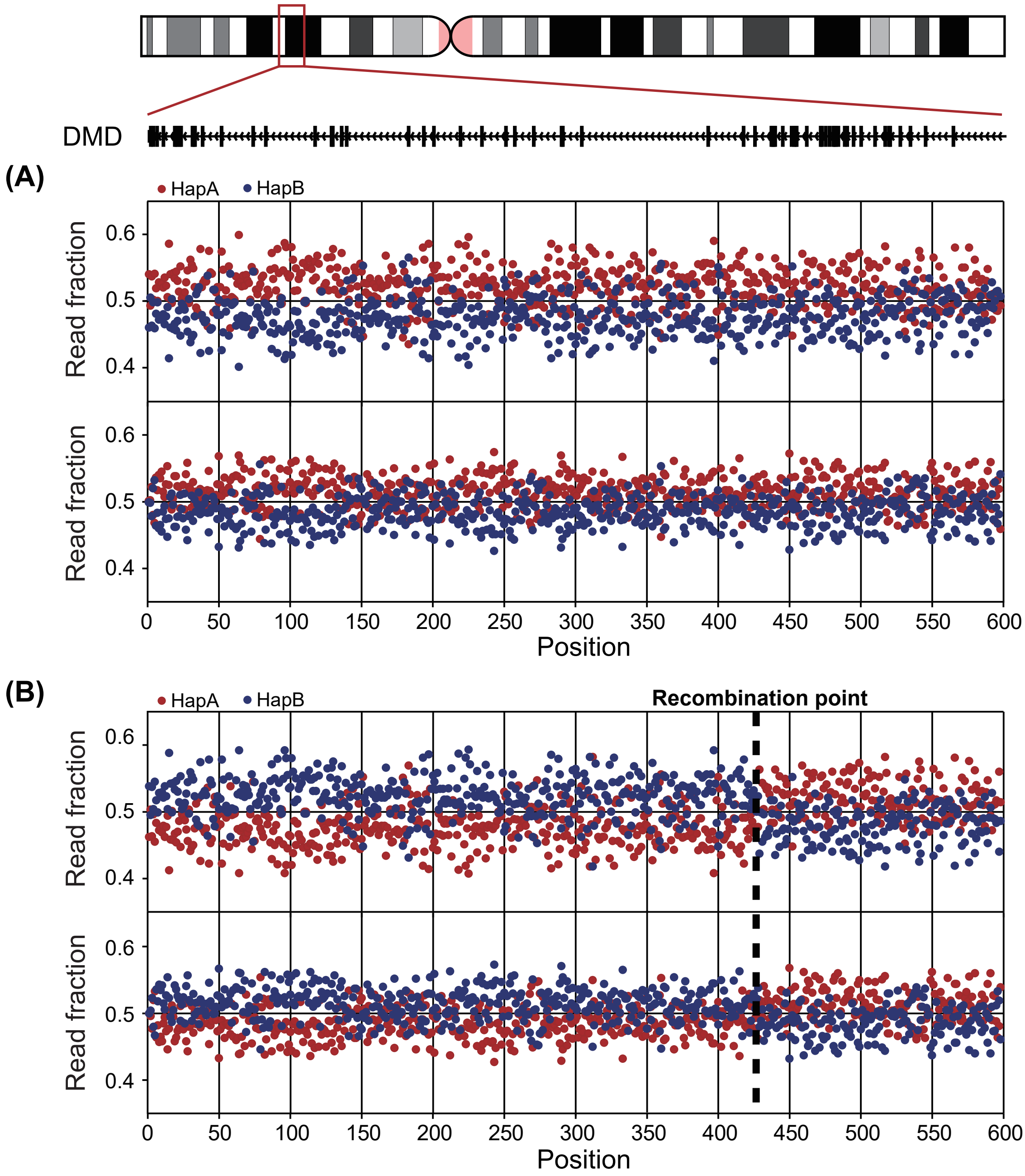

Supplementary Figure S4. Recombination detection and haplotype reconstruction in DMD-02

A. Read fraction distribution of 2 haplotypes using direct phasing. The top diagram represent plasma DNA at 9 weeks and the bottom at 12 weeks.

B. Read fraction distribution of 2 haplotypes using proband-based phasing. The black dotted line represents the recombination point predicted by the changepoint algorithm. The top diagram shows plasma DNA at 9 weeks and the bottom at 12 weeks.

Supplementary Table S1. Targeted massively parallel sequencing summary of genomic and maternal plasma DNA sequencing.

Sample	Mean molecular length (bases)	Total reads	Total reads mapped to hg19	Total reads mapped to target	Total reads mapped to target (%)	Covered bait bases >= 30 (%)	Mean depth	N50 phase block (bp)	Number of informative SNPs
DMD-01	38,135	71,808,868	71,355,903	32,197,734	44.84	99.1	686.77	41,696	740
DMD-02	34,933	79,473,835	78,592,012	33,880,770	42.63	99.1	610.28	38,361	958
DMD-03	44,252	72,921,736	72,398,702	32,158,928	44.1	99.1	751.15	46,931	705
DMD-04	27,074	75,238,466	74,692,925	34,483,239	45.83	99.1	885.97	34.619	881
DMD-05	42,809	89,246,738	88,388,188	36,186,926	40.55	99.1	527.74	51.769	730
DMD-01-fetus[a]	NA	62,140,714	61,254,461	14,857,845	23.91	99	691.7	NA	NA
DMD-02-fetus[a]	NA	53,482,735	52,826,547	11,252,767	21.04	98.9	525.8	NA	NA
DMD-03-fetus[a]	NA	33,642,168	33,334,631	7,798,255	23.18	91.5	336.13	NA	NA
DMD-04-fetus[a]	NA	29,799,572	29,478,604	7,881,987	26.45	98.4	248.04	NA	NA
DMD-05-fetus[a]	NA	29,473,959	29,411,841	6,877,801	23.38	80.3	298.49	NA	NA
DMD-01-6wk	NA	54,828,936	53,912,045	10,094,007	18.41	97.7	465.61	NA	NA
DMD-01-17wk	NA	53,776,131	52,978,358	11,454,316	21.30	98	529.38	NA	NA
DMD-02-9wk	NA	50,420,886	49,643,304	9,534,590	18.91	98	440.4	NA	NA
DMD-02-12wk	NA	65,047,893	64,180,117	17,172,644	26.40	98.6	792.31	NA	NA
DMD-03-8wk	NA	68,397,568	67,646,003	25,847,441	37.79	98.3	698.06	NA	NA
DMD-03-11wk	NA	68,874,610	68,144,635	26,096,590	37.89	98.9	724.04	NA	NA
DMD-04-7wk	NA	65,317,530	64,610,871	25,277,884	38.70	98.1	687.24	NA	NA
DMD-05-8wk	NA	69,119,036	68,947,407	24,767,584	35.83	98.9	764.24	NA	NA
DMD-05-12wk	NA	72,320,288	72,141,574	25,463,245	35.21	99.0	841.95	NA	NA

[a] Genomic DNA samples of fetuses were collected by chorionic villus sampling and amniocentesis.

Supplementary Table S2. Phasing result concurrent with the fetal genotype

	Before recombination adjustment		After recombination adjustment		
**Sample**	**Direct phasing method (%)**	**Proband-based phasing method (%)**	**Direct phasing method (%)**	**Proband-based phasing method (%)**	**Heterozygous SNVs**
DMD-01	99.46	98.92	99.46	98.92	740
DMD-02	91.65	63.05	91.65	63.05	958
DMD-03	94.61	98.01	94.61	98.01	705
DMD-04	92.62	94.67	92.62	94.67	881
DMD-05	46.30	48.49	90.82	90.27	730

Supplementary Table S3. Cost of proband-based and direct phasing methods

		Proband-based method		Direct phasing method	
	**Estimated Cost per Sample**	**Number of Samples**	**Total (USD)**	**Number of Samples**	**Total (USD)**
**gDNA QC**	30	3	90	2	60
**Sequencing library**	150	3	450	2	300
**10X library**	800	0	0	1	800
**probe capture**	500	3	1500	2	1000
**Targeted Sequencing**	100	3	300	2	200
**Total**			**$2,340**		**$2,360**
